# Cancer associated fibroblast derived gene signature determines cancer subtypes and prognostic model construction in head and neck squamous cell carcinomas

**DOI:** 10.1002/cam4.5383

**Published:** 2022-11-20

**Authors:** Sangqing Wu, Cheng Huang, Liangping Su, Ping‐Pui Wong, Yongsheng Huang, Renhui Chen, Peiliang Lin, Yuchu Ye, Pang Song, Ping Han, Xiaoming Huang

**Affiliations:** ^1^ Department of Otolaryngology Head and Neck Surgery, Sun Yat‐Sen Memorial Hospital Sun Yat‐Sen University Guangzhou China; ^2^ Guangdong Provincial Key Laboratory of Malignant Tumor Epigenetics and Gene Regulation, Guangdong‐Hong Kong Joint Laboratory for RNA medicine, Sun Yat‐sen Memorial Hospital Sun Yat‐sen University Guangzhou China; ^3^ Medical Research Center, Sun Yat‐sen Memorial Hospital Sun Yat‐sen University Guangzhou China; ^4^ The Cellular & Molecular Diagnostics Center, Sun Yat‐Sen Memorial Hospital Sun Yat‐Sen University Guangzhou China

**Keywords:** Cancer subtypes, Cancer‐Associated‐Fibroblast, Gene signature, Head and Neck Squamous Cell Carcinoma, Prognostic Model

## Abstract

**Background:**

Head and neck squamous cell carcinomas (HNSCC) are the most common type of head and neck cancer with an unimproved prognosis over the past decades. Although the role of cancer‐associated‐fibroblast (CAF) has been demonstrated in HNSCC, the correlation between CAF‐derived gene expression and patient prognosis remains unknown.

**Methods:**

A total of 528 patients from TCGA database and 270 patients from GSE65858 database were contained in this study. After extracting 66 CAF‐related gene expression data from TCGA database, consensus clustering was performed to identify different HNSCC subtypes. Limma package was used to distinguish the differentially expression genes (DEGs) between these subtypes, followed by Lasso regression analysis to construct a prognostic model. The model was validated by performing Kaplan‐Meier survival, ROC and risk curve, univariate and multivariate COX regression analysis. GO, KEGG, GSEA, ESTIMATE and ssGSEA analyses was performed to explort the potential mechanism leading to different prognosis.

**Results:**

Based on the 66 CAF‐related gene expression pattern we stratitied HNSCC patients into two previously unreported subtypes with different clinical outcomes. A prognostic model composed of 15 DEGs was constructed and validated. In addition, bioinformatics analysis showed that the prognostic risk of HNSCC patients was also negatively correlated to immune infiltration, implying the role of tumor immune escape in HNSCC prognosis and treatment option.

**Conclusions:**

The study develops a reliable prognostic prediction tool and provides a theoretical treatment guidance for HNSCC patients.

## Graphical abstract

1. High expression of cancer associated fibroblast gene signature correlates with poor prognosis in HNSCC.

2. Identified two HNSCC subtypes according to CAF‐derived gene signature expression and constructed a prognostic model on this basis.

3. The prognostic model is correlated with tumor immune microenvironment, providing a potential prognostic prediction tool and a theoretical treatment guidance for HNSCC patients.

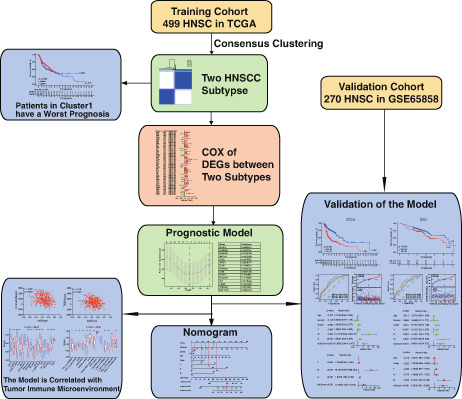

## 1 INTRODUCTION

Head and neck squamous cells carcinoma (HNSCC), accounting for more than 90% of head and neck cancer, is the seventh most common cancer worldwide, with approximately 890,000 new cases and 450,000 deaths in 2020.^1^, ^2^, ^3^ Owing to the lack of specific clinical symptoms at early stages, approximately 60% of patients present with locally advanced disease at diagnosis.^2^ The prognosis of HNSCC has not been improved in the past few decades.^4^ Besides, despite the increasing molecular biomarkers identified for HNSCC, current treatment decision and prognostic prediction are almost dependent on clinical stages. To solve these problems, it is crucial to recognize the heterogeneity of HNSCC and establish a reliable prognostic model to predict patients' clinical outcomes and guide treatment strategy.

Tumor microenvironment (TME) consists of stromal components (stromal cells, collagen, tumor vessels, etc.) and immune components (immune cells, cytokines, etc.), which is considered to be the “soil” of tumor cells and accounted for tumor heterogeneity.^5^, ^6^ Cancer‐associated‐fibroblasts (CAFs) are the most abundant stromal cells which plays a crucial part in tumor progression, invasion, metastasis, and drug resistance.^7^, ^8^ CAFs are defined as a collection of cells with multiple molecular markers, including α‐smooth muscle actin (α‐SMA) and Fibroblast activation protein (FAP), et al.^9^, ^10^, ^11^, ^12^ A meta‐analysis study indicated that the increased CAF density is correlated with clinical stages, vascular and perineural invasion, proliferation, differentiation, and local recurrence in oral squamous cells carcinoma.^13^ Besides, CAFs were confirmed to mediate IL‐33/CXCR4 signaling circuit to promote the invasiveness of HNSCC,^14^ highlighting a vital role of CAFs in HNSCC. Nevertheless, the association between CAF derived gene signature expression pattern and its functional roles in HNSCC remains unexploited.

In this work, we sought to examine the expression pattern of CAF‐related gene signature in HNSCC, which in turn was used for constructing a model for HNSCC prognostic prediction and treatment guidance. We first selected 66 CAF‐related genes from literature studies^11^, ^14^, ^15^, ^16^, ^17^, ^18^, ^19^, ^20^, ^21^, ^22^, ^23^, ^24^, ^25^, ^26^, ^27^, ^28^, ^29^ and downloaded their expression and clinical data from TCGA and GEO databases. Further consensus clustering data analysis of CAF‐derived gene signature expression data identified two new HNSCC subtypes (i.e. cluster 1 and 2), indicating that HNSCC patients assigned to cluster 1 had more stromal infiltration and poor survival as compared to the patients in cluster 2. By analyzing the prognosis‐related differentially expression genes (DEGs) between these subtypes, our work showed that 15 of these genes' expression were sufficient to be used for constructing an effective prognostic model for HNSCC. Additionally, further pathway enrichment analysis showed that the patient prognosis was negatively associated with immune infiltration, providing a theoretical guidance for the formulation of treatment strategies in HNSCC.

Overall, our findings identify two previously unidentified HNSCC subtypes with different clinical performances and construct an effective prognostic model by which we can predict patients' prognosis and potentially develop more appropriate treatment plans. With the used of this model, we can predict the prognostic risk of patients and adjust the treatment plan in time, which is expected to improve the prognosis of patients with HNSCC.

## 2 MATERIALS AND METHODS

### 2.1 Transcriptome RNA‐sequence data and clinical data analysis

Transcriptome RNA‐sequence data of 528 HNSCC cases and paired clinical data were obtained from The Cancer Genome Atlas (TCGA) database (https://portal.gdc.cancer.gov/) as the training cohort, RNA‐sequence and clinical data of GSE65858 including 270 HNSCC cases were downloaded from the Gene Expression Omnibus (GEO) database (https://www.ncbi.nlm.nih.gov/geo/) as the validation cohort. CAF‐related genes were obtained based on literature study.

### 2.2 Consensus clustering analysis

The expression of 66 CAF‐related genes were extract from the training cohort and then we performed consensus clustering using the R package “ConsensusClusterPlus” to classify HNSCC patients according to the above 66 signatures. The cumulative distribution function (CDF) curve and the relative change of the area under CDF curve were used to determine the most reliable cluster number.

### 2.3 Tumor microenvironment (TME) analysis

To assess the component of TME, the ESTIMATE algorithm was performed using the R package “estimate”.^30^ StromalScore, ImmuneScore, TMEScore refer to the ratio of stromal components, immune components, and the sum of both respectively.

### 2.4 Differentially expressed genes (DEG) analysis

Tumor samples were labeled with cluster 1 or cluster 2 according to the clustering, and R package “limma” was used to analysis the differentially expressed genes (DEGs) between two cluster. DEGs with fold change >1.5 and FDR <0.05 were considered significant. R package “Pheatmap” was used to draw the heatmap of DEGs.

### 2.5 Survival analysis and construction of prognostic model

R package “survival” was used to perform univariate COX regression analysis to extract DEGs related to patient prognosis (*p* < 0.05). Subsequently, those DEGs were included to construct a Least Absolute Shrinkage and Selection Operator (Lasso) regression model using the R package “glmnet” and “survival”. This model presents patients' prognostic status by risk score, according to the formula as follow: RiskScore = Coef1*DEG1exp + Coef2*DEG2exp + … + Coefi*DEGiexp

### 2.6 Evaluation of prognostic model

Patients on raining cohort and validation cohort were divided into high‐risk group and low‐risk group respectively based on the median cutoff risk score of validation cohort, and then Kaplan–Meier survival analysis was performed using R package “survival” and “survminer” to verify the prognosis between two groups. 1‐, 3‐, 5‐year ROC curves were drawn to evaluate the sensitivity and specificity of the model using R package “timeROC”. The risk curve was drawn using R package “pheatmap” to show the correlation between each patients' risk score and survival status. Univariate and multivariate COX regression was utilized for independent prognostic analysis.

### 2.7 Construction of nomogram

To predict the 1‐, 3‐, and 5‐year survival rate of HNSCC patients more conveniently and accurately, the risk score along with patient's characteristic, including age, gender, stage, T stage, N stage, M stage, were contained to draw a nomogram using R package “rms”. The calibration curves were drawn to assess the accuracy of the nomogram.

### 2.8 GO, KEGG and GSEA analysis

A series of enrichment analyses were performed to explore the potential biological functions and signaling pathways between different groups. Gene Ontology (GO) and Kyoto Encyclopedia of Genes and Genomes (KEGG) enrichment analysis were performed using R package “clusterProfiler”, “enrichplot” and “ggplot2”. Gene Set Enrichment Analysis (GSEA) was performed via GSEA_4.0.3 software.

### 2.9 Single sample gene set enrichment analysis

Single Sample Gene Set Enrichment Analysis(ssGSEA)was applied to compare the amount of immune cells and the activity of immune‐related processes, using R package “GSVA”, “GSEABase”, and “limma”.

### 2.10 Statistical analysis

R studio version 4.0.1 and GraphPad Prism version 9 were utilized for all statistical analyses. The Kaplan–Meier method was used to analyze the survival between two subtypes. The correlations between risk score and TME components were analyzed by Pearson correlation. *p* < 0.05 was considered as significance.

## 3 RESULTS

### 3.1 Analysis process of this study

**FIGURE 1:**
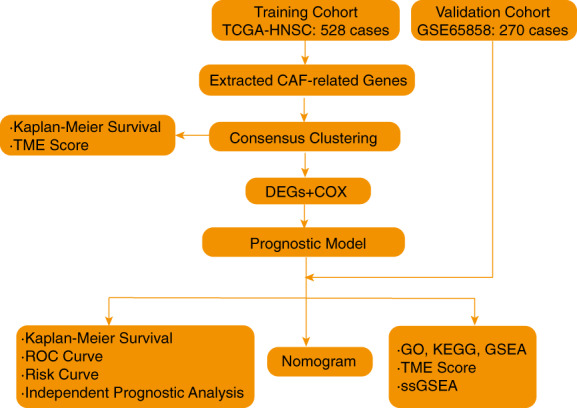
Analysis process of this study.

The flow chart of our study is shown in Figure 1. We first downloaded the transcriptome RNA‐sequencing data of 528 HNSCC cases from TCGA database (as a training cohort) (Table S1) and extracted the expression data of 66 CAF‐derived genes out of it (Table S2). Subsequently, consensus clustering was performed to identify two new subtypes of HNSCC. The DEGs of these two subtypes related with patient prognosis were selected to construct a prognostic model. To validate our model, we downloaded the RNA‐sequencing data of 270 HNSCC cases from GSE65858 database (Table S3) (as a validation cohort), which was then subjected for Kaplan–Meier survival, ROC curve and risk curve as well as independent prognostic analysis to validate the prognostic model. Additionally, a nomogram was constructed to visualize this model. Finally, followed by dividing patients into high and low prognostic risk groups according to the prognostic model, we performed a comprehensive GO, KEGG and GSEA analysis to explore the differences between two risk groups, while tumor microenvironment (TME) score and single‐sample gene set enrichment analysis (ssGSEA) was also used to explore the correlation between risk score and tumor microenvironment.

### 3.2 Identification of two previously unreported HNSCC subtypes based on CAF‐derived gene signature expression

**FIGURE 2:**
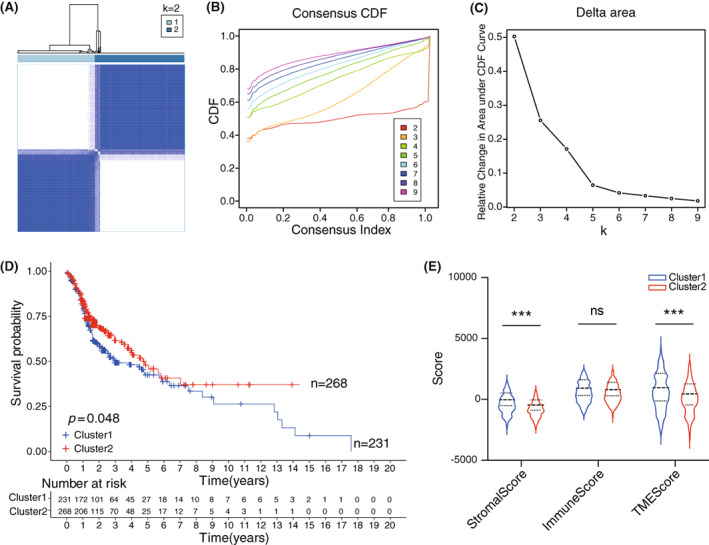
Identification of two HNSCC subtypes based on CAF‐related gene signature expression. (A) Consensus matrix of HNSCC patients (*n* = 499). (B) Cumulative distribution function. (C) The relative change of the area under CDF curve. (D) Kaplan–Meier curve indicated that patients of cluster 1 have a worse prognosis, *p* = 0.048 by log‐rank test (Cluster1, *n* = 231, Cluster2, *n* = 268). (E) StromalScore and TMEScore were significantly higher in cluster 1 than cluster 2, while ImmuneScore was equivalent between two clusters. **p < 0.001. ns, no significant difference. Student's *t*‐test.

Based on the 66 CAF‐related gene expression pattern in our training cohort, we applied consensus clustering algorithm to stratify HNSCC patients. According to the cumulative distribution function (CDF) curve and the relative change of the area under CDF curve, k = 2 was the most reliable cluster number (Figure 2B,C), which could divide patients into two clusters: cluster 1 and 2 (Figure 2A). Kaplan–Meier survival analysis showed poor prognosis of patients in cluster 1 as compared to cluster 2 (Figure 2D, *p* = 0.048). We also analyzed DEGs between two groups and subsequently performed KEGG, GO, and GSEA analyses to explore the potential functional differences. The results indicated that DEGs were mainly enriched in muscle development and differentiation terms (Figure S1A,B). Further GSEA analysis displayed that the enriched genes in the cluster 1 were related to myogenesis, apical Junction, epithelial meschymal transition (EMT) and coagulation pathways, while the genes in the cluster 2 were mainly enriched in MYC targets, oxidative phosphorylation, E2F targets and DNA repair pathways (Figure S1C,D).

To compare the proportion of TME between two clusters, we applied ESTIMATE algorithm and found that the ratio of stromal components in the cluster 1 was significantly higher than in the cluster 2 (*p* < 0.001). Despite the difference of ImmuneScore between two clusters had no statistical significance (*p* = 0.180), the TMEScore in the cluster 1 was still significantly higher than that in the cluster 2 (*p* < 0.001) (Figure 2 E). Overall, our work discovered two previously unidentified HNSCC subtypes, indicating that the patients assigned to the cluster 1 had a worse prognosis compared to the patients in the cluster 2, which might be caused by the infiltration of TME.

### 3.3 Construction of a 15‐gene signature prognostic model for HNSCC

**FIGURE 3:**
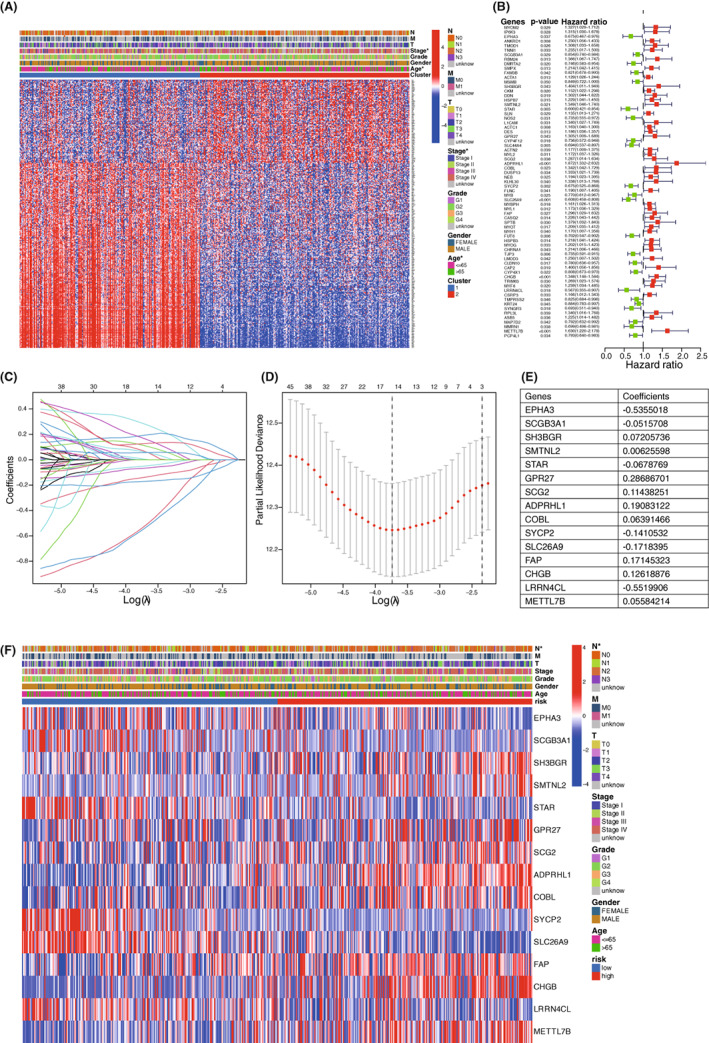
Construction of a 15‐gene signature prognostic model for HNSCC. (A) Heatmap of DEGs between two clusters. (B) Forest plot showed DEGs related with patients' survival status. (C) Lasso regression model. (D) Cross validation showed that 15 was the optimum λ value. (E) Table of 15 genes and their corresponding coefficients included in the prognostic model. (F) Heatmap of 15 genes' expression in two risk groups and the relationship between risk score and clinical features was shown repsectively. *p < 0.05. Chi‐square test.

We next sought to identify the prognosis‐related genes of HNSCC patients in order to construct a prognostic model. Limma package was used to analyze the DEGs between two clusters (Figure 3A), followed by COX regression analysis to screen genes related with prognostic status (Figure 3B). These genes were contained to perform Lasso regression, and a 15‐genes model was established according to the optimum λ (Figure 3C–E). The risk score could be calculated as following formula:

RiskScore = −0.54*EPHA3 exp −0.05*SCGB3A1 exp +0.07*SH3BGR exp +0.01*SMTNL2 exp −0.07*STAR exp +0.29*GPR27 exp +0.11*SCG2 exp +0.19*ADPRHL1 exp +0.06*COBL exp −0.14*SYCP2 exp −0.17*SLC26A9 exp +0.17*FAP exp +0.13*CHGB exp −0.55*LRRN4CL exp +0.06*METTL7B exp.

According to the formula, we calculated patients' risk score and assigned them into high risk or low risk group based on the median cutoff score. Importantly, we then performed differential analysis by using limma package, which showed higher expression of *SH3BGR*, *SMTNL2*, *GPR27*, *SCG2*, *ADPRHL1*, *COBL*, *FAP*, *CHGB*, *METTL7B* and lower expression of *EPHA3*, *SCGB3A1*, *STAR*, *SYCP2*, *SLC26A9*, and *LRRN4CL* in high risk group as compared to low risk group (Figure 3F). We further examined the correlation between risk score and clinical characteristics, indicating that the risk score was independent of clinical features including age, gender, grade, stage, tumor (T) stage, and distant metastasis (M) status, whereas high risk score was associated with increased lymph node metastasis (*p* = 0.032). Overall, our work built up a 15‐gene signature prognostic model of HNSCC patients.

### 3.4 Combined Kaplan–Meier survival curve, ROC curve, risk curve and independent prognostic analysis indicates a good performance of the HNSCC prognostic model

**FIGURE 4:**
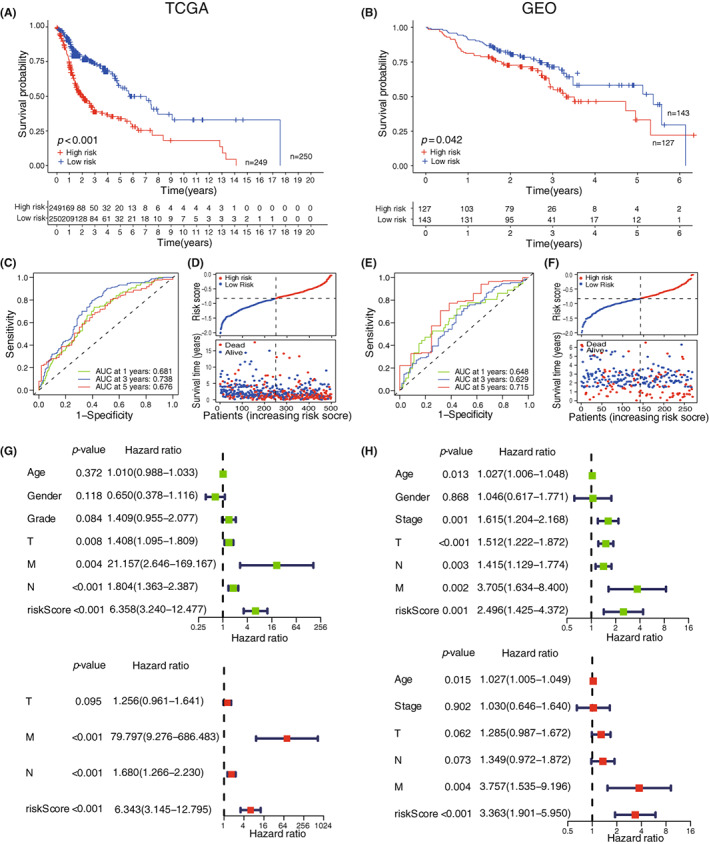
Combined Kaplan–Meier curve, ROC curve, risk curve and independent prognostic analysis demonstrates a good performance of the HSNCC prognostic model. (A, B) Kaplan–Meier curve showed that patients in the high risk group was associated with poor prognosis in both training (*n* = 499 patients) (A) and validation cohorts (*n* = 270 patients) (B). (C, E) ROC curves of the training cohort (C) and validation cohort (E). (D, F) Risk curve showed that the number of deaths (bottom, red dots) increased as the risk score increased (top) in both training (D) and validation cohorts (F). (G, H) Univariate COX regression (top) and multivariate COX regression (bottom) indicated risk score as an independent prognostic factor in both training (G) and validation cohorts (H). AUC, Area Under Curve.

To evaluate the efficacy of the model above, we downloaded RNA‐sequencing and clinical data of 270 HNSCC cases from GEO dataset (GSE65858) and used the data as a validation cohort. According to the prognostic model, we first calculated the risk score of each patient and then allocated them into high risk or low risk group based on the median cutoff risk score of the training cohort. By performing Kaplan–Meier survival analysis, our data indicated that the patients assigned to high risk group had a worse prognosis in both training (*p* < 0.001, Figure 4A) and validation (*p* = 0.042, Figure 4B) cohorts. Further ROC curve analysis showed good specificity and sensitivity of this risk model, with 1‐, 3‐, 5‐year survival area under curve (AUC) of 0.681, 0.738, 0.676 in the training cohort (Figure 4C) and 0.648, 0.629, 0.715 in the validation cohort respectively (Figure 4 E). In addition, the risk curve was drawn to reflect the changes in patients' survival status according to their risk score intuitively. The result suggested that the number of deaths was increased as the risk score increased both in training (Figure 4D) and validation (Figure 4F) cohorts.

Furthermore, the risk score was identified as an independent prognostic indicator according to univariate COX regression (training cohort, HR = 6.358, 95% CI: 3.240–12.477, *p* < 0.001; validation cohort, HR = 2.496, 95% CI: 1.425–4.372, *p* = 0.001) and multivariate COX regression (training cohort, HR = 6.343, 95% CI: 3.145–12.795, *p* < 0.001; validation cohort, HR = 3.363, 95% CI: 1.901–5.950, *p* < 0.001) (Figure 4G,H). Collectively, these results confirmed that our model had good capability and accuracy in predicting HNSCC patient prognosis.

### 3.5 Construction of a nomogram

**FIGURE 5:**
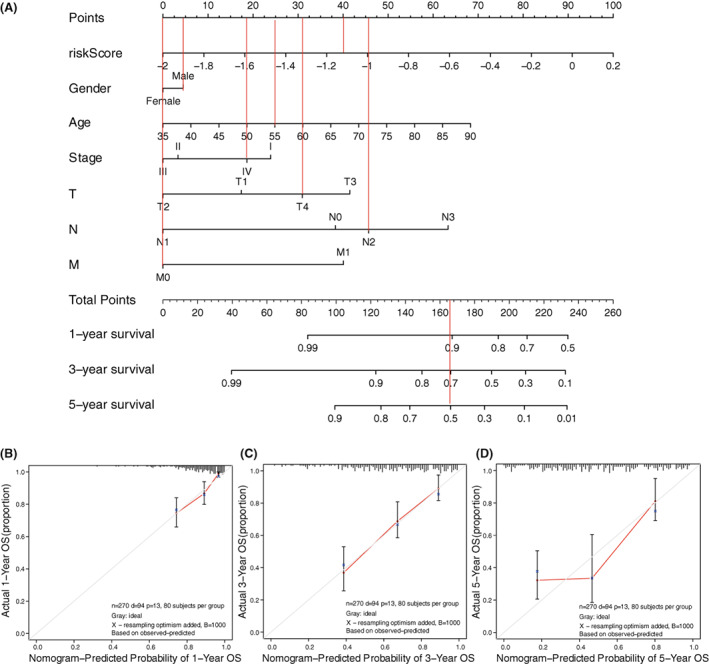
Construction of a Nomogram. (A) A nomogram containing risk score to predict 1‐, 3‐, 5‐year survival of HNSCC patients. (B–D) 1‐year (B), 3‐year (C), 5‐year (D) surivval calibration plots showed that the prediction calibration curve (red) was close to the standard curve (gray), verified a good predictive ability of the nomogram. OS, Overall Survival.

To predict HNSCC patients' survival more conveniently and precisely, we established a nomogram (Figure 5A). For example, a 55‐year‐old male HNSCC patient, T4N2M0, Stage IV, with a risk score of −1.16 according to the prognostic model. We got points according to each characteristic and then added them up to get the total point, which predicted that this patient's 1‐, 3‐, 5‐year survival was about 90%, 70% and 50% respectively. Importantly, the calibration plots for 1‐, 3‐, 5‐year survival verified good predictive ability of the nomogram (Figure 5B–D).

### 3.6 Correlation between prognostic risk and tumor immune microenvironment

To further explore the potential mechanisms leading to different risk scores, we used limma package to analysis the DEGs between two risk groups in the training cohort, followed by KEGG and GO enrichment analysis. KEGG analysis suggested that the DEGs were significantly enriched in metabolic and immune pathways such as tyrosine and pyruvate metabolism, fatty acid degradation and primary immunodeficiency (Figure 2A). GO analysis showed that the DEGs were associated with keratinocyte development and receptor ligand activity (Figure 2B). In addition, GSEA indicated that high risk group mostly enriched in tumor promoting gene sets (Figure 2C). Interestingly, our results showed that low risk group notably enriched in various immune pathways (Figure 2D,E), such as T cell receptor signaling pathway, B cell receptor signaling pathway, primaty immunodeficiency, and chemokine signaling pathway, suggesting that it may be associated with immune activation/response in these patients.

**FIGURE 6:**
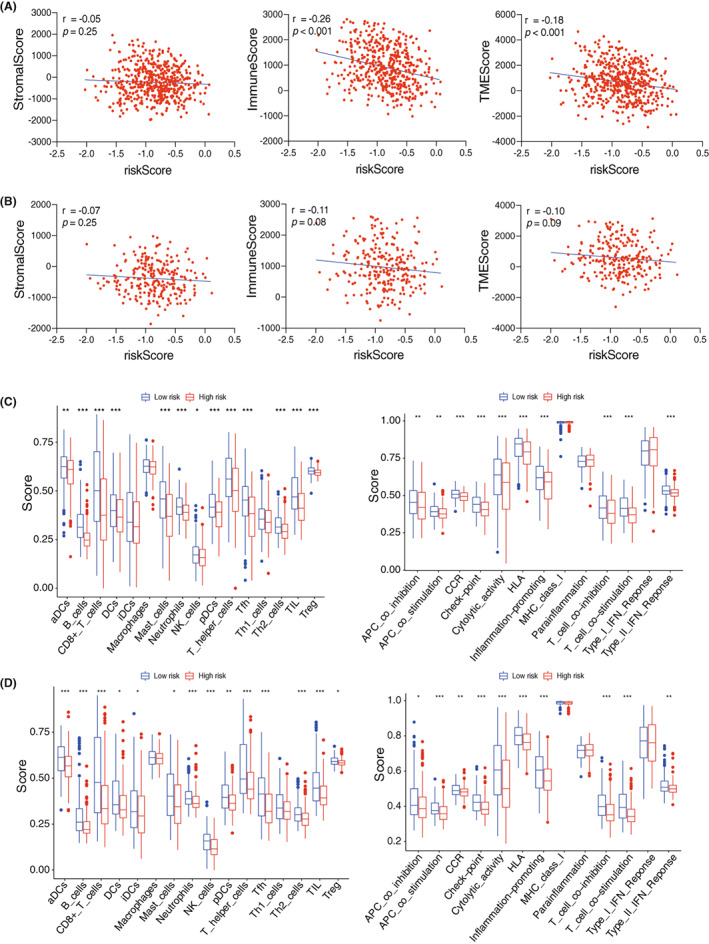
Correlation Between Prognostic Risk and Tumor Immune Microenvironment. (A, B) The correlation between risk score and StromalScore/ImmuneScore/TMEScore in training cohort (A) or validation cohort (B) is given as indicated. (C, D) The number of immune cells (top) and the activity of immune‐related processes (bottom) in training cohort (C) and validation cohort are shown (D).

Hence, we next focused on the correlation between risk score and tumor immune microenvironment. Pearson correlation analysis showed that the immune score was negatively correlated with risk score (*r* = −0.26, *p* < 0.001) in training cohort (Figure 6A), which was consistent with GSEA results above. There was also a negative correlation between immune score and risk score in validation cohort (Figure 6B). Furthermore, ssGSEA analysis was performed to work out the number of immune cells and the activity of immune‐related processes, indicating that they were both higher in low risk group as compared to high risk group (Figure 6C), which was further confirmed in the validation cohort (Figure 6D). Overall, our results suggested that the potential mechanisms contributed to high prognostic risk might be related to the activation of tumor promoting pathways and the suppression of immune‐related processes in HNSCC patients.

## 4 DISCUSSION

Head and neck squamous cells carcinomas are the most common tumors in head and neck.^31^ Despite the major advance in minimally invasive surgery, radiochemotherapy, and immunotherapy, the survival of patients has not been significantly improved over the past decades.^4^, ^32^ The lack of reliable prognostic biomarkers and tumor heterogenicity causes complications and difficulties in HNSCC treatment, which in turn contributes to its unimproved prognosis. Hence, it is a clinical unmet need to establish an efficient prognostic model for HNSCC patients. In this study, our work demonstrated for the first time that the expression of CAF‐derived gene signature was able to stratify HNSCC into two new subtypes with different clinical outcomes, which could be used to construct an effective 15‐gene signature prognostic model for HNSCC patients.

Tumor microenvironment (TME) plays an important role in cancer occurrence and development, which is mainly due to the crosstalk between cancer and stromal cells.^33^, ^34^, ^35^ Yoshihara K et al. developed the ESTIMATE algorithm to evaluate the infiltration of stromal and immune cells in TME using gene expression data, showing that the level of stromal and immune cells in TME might be correlated with patients' clinical characteristics.^30^ Amonst all stromal cells, CAFs are considered as the most important ones because of their abundance and their strong interaction with cancer cells.^7^, ^35^ CAFs were proven to promote the invasion and treatment resistance of cancer cells through paracrine and extracellular matrix (ECM) remodeling in many solid tumors including HNSCC,^36^, ^37^, ^38^, ^39^, ^40^ however, the clinical significance of CAF‐related genes expression remains unclear. In our study, we identified two new HNSCC subtypes based on the 66 CAF‐related genes expression level obtained from TCGA database, while further consensus clustering analysis showed that the patients assigned to the cluster 1 had higher stromal infiltration and worst prognosis than the patients in the cluster 2. Consistent with this finding, our enrichment analysis indicated that the patients in the cluster 1 mostly enriched in pathways associated with ECM, such as myogenesis and EMT. In summary, our bioinformatic analysis successfully utilized CAF‐associated gene expression signature to identify two new HNSCC subtypes, while this classification associated with patients' prognosis and also correlated with tumor microenvironment status, suggesting the potential significance of CAFs in HNSCC patients' prognosis.

Since the prognostic value of CAF‐related genes was uncovered in this study, we further explored their potential to be used for constructing a HNSCC prognostic model. By screening the prognosis associated DEGs of the two newly identified subtypes using COX regression, we successfully established a prognostic risk model based on 15 of those genes. Moreover, we downloaded another dataset from GEO database as a validation cohort and verified the good efficacy of the model. Firstly, we divided patients into high risk or low risk group according to the median cutoff risk score calculated by the formula of the risk model and proved that the patients in high risk group had a worst prognosis as compared to the patients in low risk group. Secondly, the ROC curve showed a high specificity and sensitivity of the model, while the AUC of 1‐, 3‐, and 5‐year survival were all larger than 0.65. The risk curve visually showed that the number of patient deaths increased while the risk score increased. Critically, by perfoming univariate and multivariate COX regression analysis, we confirmed the risk score as an independent prognostic indicator. Finally, our work established a prognostic predicted nomogram including a risk score and other clinical features for potential clinical application. Overall, we successfully constructed a prognostic model for HNSCC patients based on two newly identified subtypes above, therefore providing guidance for patients' treatment in a time effective manner.

We next sought to explore the underlying mechanisms leading to different prognostic risk. Interestingly, our work discovered that low risk group was closely associated with immune pathways, while GSEA analysis showed that low risk group mostly enriched in immune pathways such as T cell receptor signaling pathway, B cell receptor signaling pathway, primary immunodeficiency, and chemokine signaling pathway, which indicated an immunologically active state in the low risk group, while on the contrary, an immunosuppressed state in the high risk group was observed. Tumor immune microenvironment, including various immune cells, cytokines, surface receptors, etc., is a dynamically changing environment, whose diversity and complexity determine tumor proliferation, invasion, metastasis, and response to immunotherapy.^41^ It's generally considered that in the early stages of tumors, immune cells and related stromal components recruited and activated by tumor cells can form a tumor‐inhibiting inflammatory microenvironment to hinder tumor development.^42^ However, with the exhaustion of killer immune cells and the recruitment of inhibitory immune cells, a immunosuppressive microenvironment is formed, which can promote tumor immune escape, thereby increasing tumor invasion and antagonistic treatment.^42^ It is reported that immunotherapy such as nivolumab may be particularly effective in tumors with high levels of infiltrating lymphocyte, making ESTIMATE immune score a potential biomarker for immunotherapy response.^30^ To further confirm our finding, we calculated the correlation between patients' risk score and TME score, indicating that the immune score was significantly negatively correlated with risk score. In addition, ssGSEA identified a significant lower number of immune cells, such as CD8+ T cells, Dendritic cells, NK cells, and poor activity of immune‐related processes such as chemokine receptor activity and immune checkpoint activity for patients in high risk group compared to those in low risk group, suggesting the presence of immunosuppressive TME in the high risk group. Overall, our research demonstrated, for the first time, that the patients' prognosis risk was strongly associated with TME status in HNSCC, suggesting that those patients in the low risk group could be more sensitive to immunotherapy as compared to the patients in high risk group, while alternative treatments should be given to the patients in high risk group.

There are several concerns remained to be addressed in our study. Firstly, although we validated the prognostic model with a cohort from GEO database, an external clinical cohort is needed to verify the predictive value in practice. Besides, since tumor immune microenvironment includes different types of immune cells and cytokines, further investigation is required to figure out the exact type of cell and pathway that mostly contributes to patients' prognosis, therefore we can formulate more effective treatment strategies.

In conclusion, our study identifies two unreported HNSCC subtypes according to CAF‐related gene signature expression and constructs a prognostic model on this basis. Importantly, the prognostic model is found to be correlated with tumor immune microenvironment, providing us a potential prognostic prediction tool and a theoretical treatment guidance for HNSCC patients. With the used of this model, we can predict the prognostic risk of patients and adjust the treatment plan in time, which is expected to improve the prognosis of patients with HNSCC.

## AUTHOR CONTRIBUTIONS

**Sangqing Wu:** Formal analysis (equal); methodology (equal); resources (equal); writing – original draft (equal). **Cheng Huang:** Data curation (equal); software (equal); validation (equal). **Liangping Su:** Supervision (equal); validation (equal). **Ping Pui Wong:** Conceptualization (equal); writing – review and editing (equal). **Yongsheng Huang:** Formal analysis (equal); software (equal); validation (equal); writing – review and editing (equal). **Renhui Chen:** Supervision (equal); validation (equal); visualization (equal). **Peiliang Lin:** Methodology (equal); project administration (equal); software (equal). **Yuchu Ye:** Formal analysis (equal); software (equal). **Pang Song:** Data curation (equal); investigation (equal). **Ping Han:** Supervision (equal). **Xiaoming Huang:** Conceptualization (equal); supervision (equal); writing – review and editing (equal).

## FUNDING INFORMATION

This work was supported by the grant [2013] 163 from the Key Laboratory of Malignant Tumor Molecular Mechanism and Translational Medicine of the Guangzhou Bureau of Science and Information Technology Grant KLB09001 from the Key Laboratory of Malignant Tumor Gene Regulation and Target Therapy of the Guangdong Higher Education Institutes. This work was also supported by the grants from the National Natural Science Foundation of China (No. 81872193, 81,702,697), the Sun Yat‐Sen University Clinical Research 5010 Program (No. 2010008), the Natural Science Foundation of China (81,920,108,028 and 81,872,142), Guangzhou Science and Technology Program (201904020008), Guangdong Science and Technology Department (2020A0505100029, 2020B1212060018, and 2020B1212030004).

## CONFLICT OF INTEREST

The authors declare that they have no competing interests.

## ETHICS APPROVAL STATEMENT

Not applicable.

## PATIENT CONSENT STATEMENT

Not applicable.

## PERMISSION TO REPRODUCE MATERIAL FROM OTHER SOURCES

Not applicable.

## CLINICAL TRIAL REGISTRATION

Not applicable.

## Supporting information


Figure S1

Figure S2

Table S1

Table S2

Table S3

## Data Availability

The datasets generated and/or analyzed during the current study are available in the TCGA repository, https://portal.gdc.cancer.gov/, and GEO repository, https://www.ncbi.nlm.nih.gov/geo/.
