# Supplementary figures and images for: Cancer associated fibroblast derived gene signature determines cancer subtypes and prognostic model construction in head and neck squamous cell carcinomas
Source: Cancer Med. 2022 Nov 20;12(5):6388–400. doi: 10.1002/cam4.5383 (PMC10028128; doi:10.1002/cam4.5383)

# Supplementary Figure 1

A

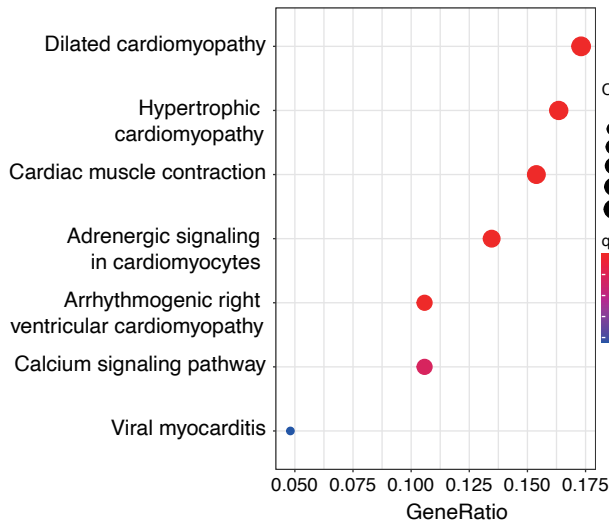

B

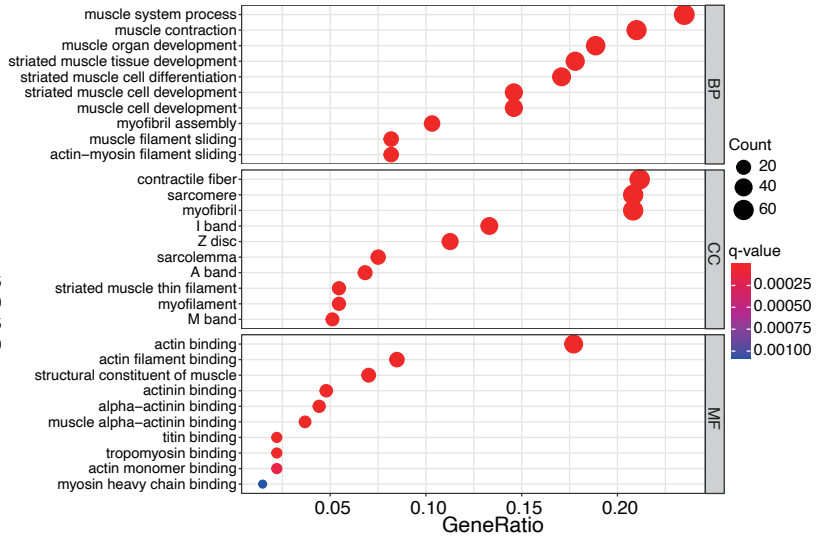

C

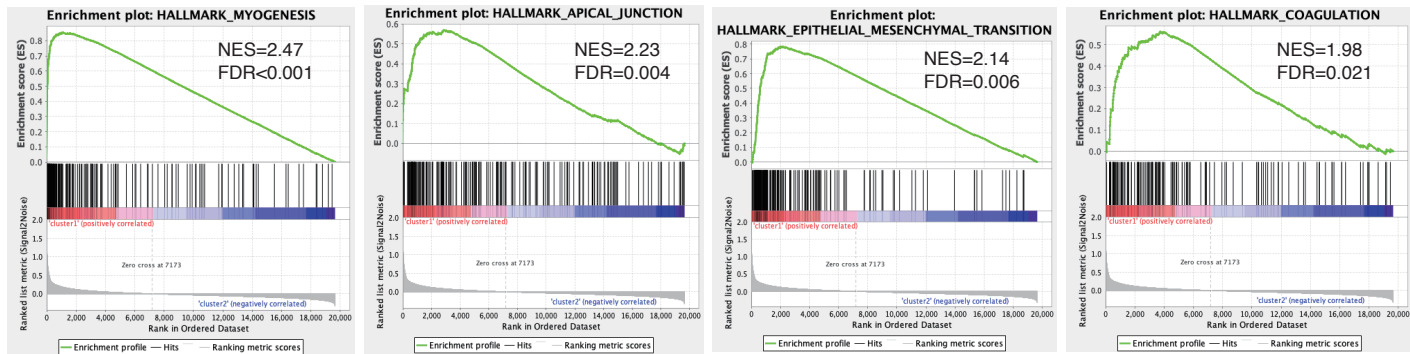

D

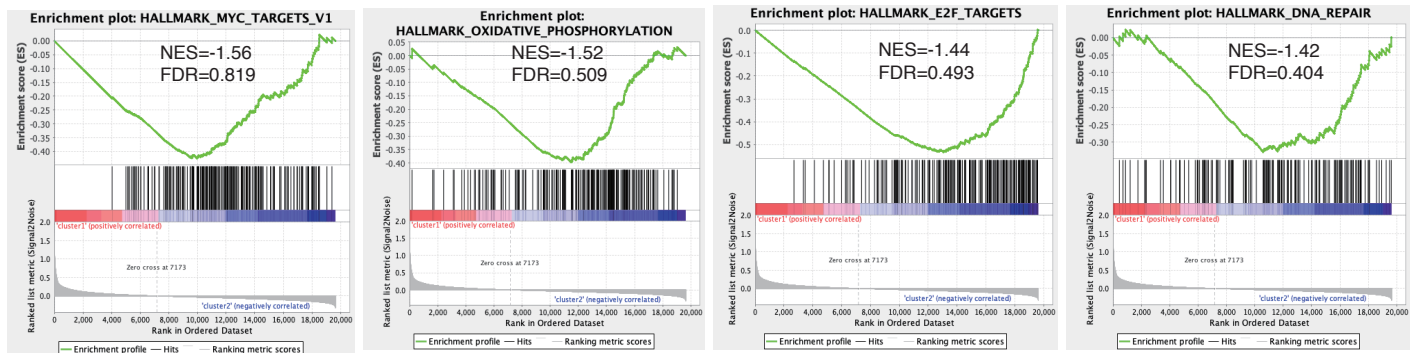

Supplement: Supplementary file 1 — Figure S1 [file CAM4-12-6388-s005.pdf]

# Supplementary Figure 2

A

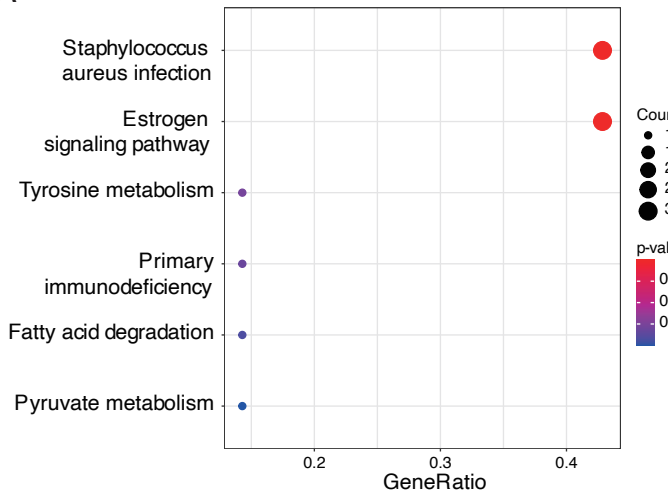

B

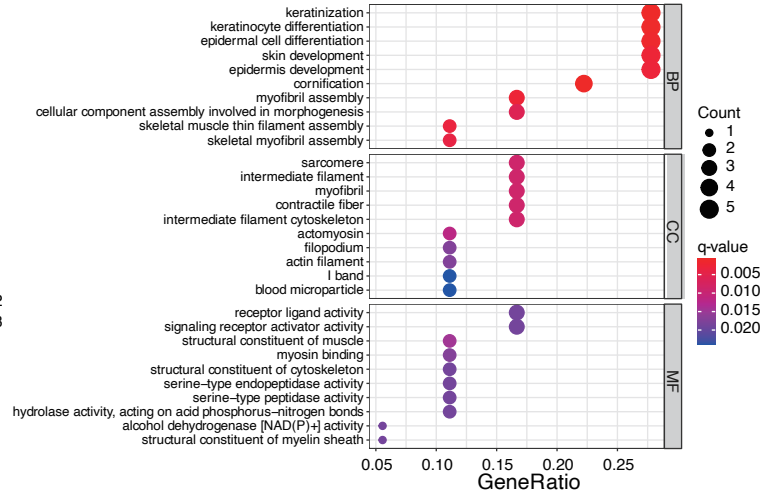

C

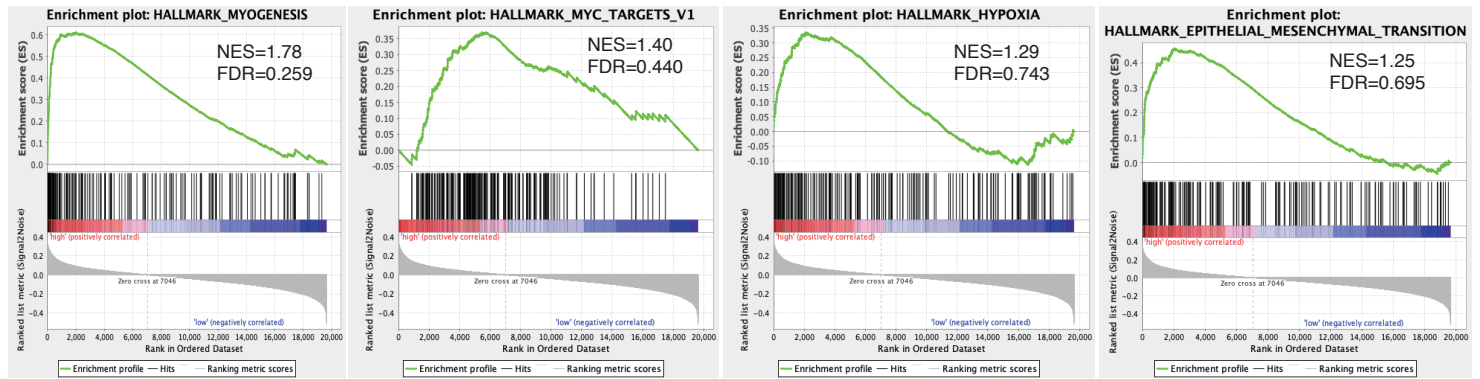

D

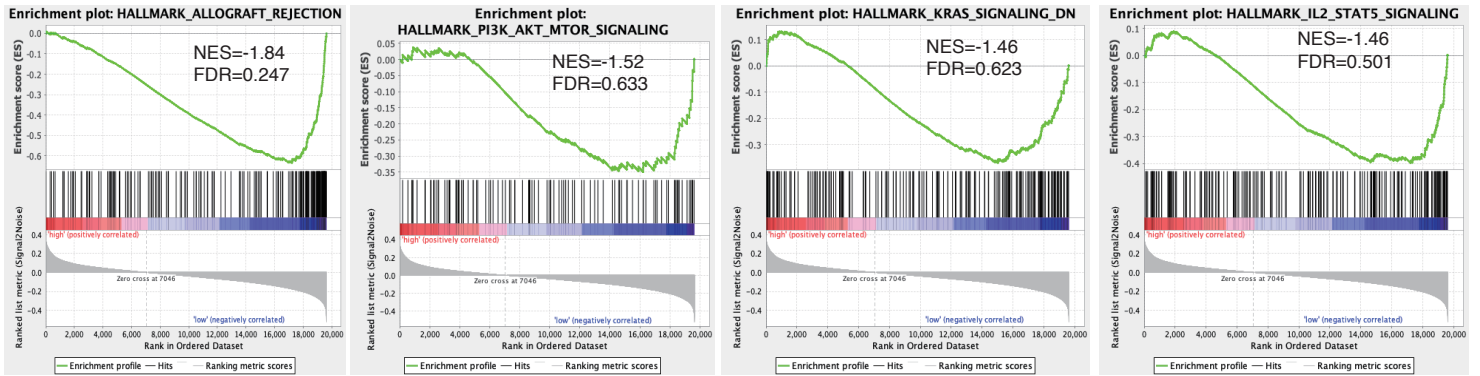

E

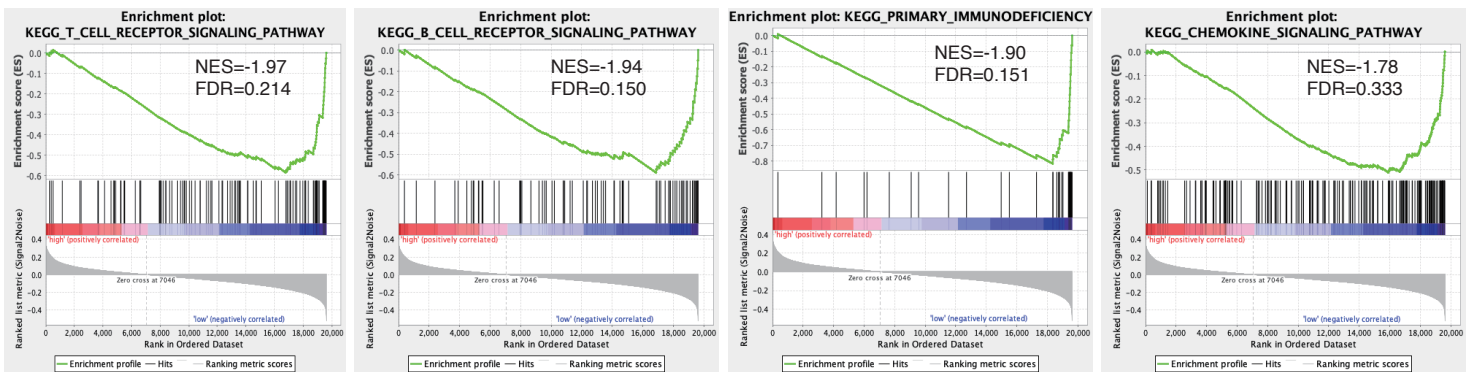

Supplement: Supplementary file 2 — Figure S2 [file CAM4-12-6388-s001.pdf]
