# Supplementary material for: Cancer associated fibroblast derived gene signature determines cancer subtypes and prognostic model construction in head and neck squamous cell carcinomas
Source: Cancer Med. 2022 Nov 20;12(5):6388–400. doi: 10.1002/cam4.5383 (PMC10028128; doi:10.1002/cam4.5383)
Supplement: Supplementary file 3 — Table S1 [file CAM4-12-6388-s004.docx]

**Supplementary Table 1 Characteristics of HNSCC patients from TCGA database**

| Characteristics | Cases（%） |
| --- | --- |
| Age  <65  ≥65 | 330（62.5%）  198（37.5%） |
| Gender  male  female  Tumor Site  oral  oropharynx  larynx  hypopharynx  unknown  Stage  I  II  III  IV  unknown  T Stage  T1  T2  T3 | 386（73.1%）  142（26.9%）  330（62.5%）  49（9.3%）  112（21.2%）  10（1.9%）  27（5.1%）  27（5.1%）  74（14.0%）  82（15.5%）  270（51.2%）  75（14.2）  49（9.3%）  140（26.5%）  101（19.1%） |
| T4  TX  N Stage  N0  N1  N2  N3  NX  M Stage  M0  M1  MX | 175（33.2%）  63（11.9%）  180（34.1%）  68（12.9%）  172（32.6%）  8（1.5%）  100（18.9%）  191（36.2%）  1（0.2%）  336（63.6%） |
