# Supplementary material for: Cancer associated fibroblast derived gene signature determines cancer subtypes and prognostic model construction in head and neck squamous cell carcinomas
Source: Cancer Med. 2022 Nov 20;12(5):6388–400. doi: 10.1002/cam4.5383 (PMC10028128; doi:10.1002/cam4.5383)
Supplement: Supplementary file 4 — Table S2 [file CAM4-12-6388-s003.docx]

**Supplementary Table 2 66 Cancer associated fibroblast-related genes**

| Genes | Cancer Type | Reference |
| --- | --- | --- |
| ACTA2、FAP、FSP1、S100A4、VIM、COL1A2、COL1A1、DDR2、DES、ITGA1、P4HA3、LUM、DCN、PDPN  CAV1、TGF  IFN  HGF、FGF1-12、FGFR1-4、SMAD2  IL33、CXCR4  CD10、GPR77、IL-6、IL-8、NFKB | Pan-cancer  Head and Neck Cancer  Breast Cancer  Lung Cancer  Head and Neck Cancer  Breast and Lung Cancer | Qian et al., 2020  Hu et al., 2021  Zhu et al., 2021  Broad et al., 2021  Hu et al., 2021  Lin et al., 2021  Su et al., 2019 |
| PDGFRA、PDGFRB、SOX9、SCRG1  VEGF、EGF  CCL5、TNF、CXCL8  CD29  TAGLN、TNC、Ly6C、C3、CXCL12、LRCC15  CD34、CTGF、CXCL12、TNC、RGS5 | Breast Cancer  Cholangiocarcinoma  Breast Cancer  Breast Cancer  Pancreatic Cancer  Melanoma | Valdes et al., 2019  Cadamuro et al., 2019  Liubomirski et al., 2019  Costa et al., 2018  Biffi et al., 2019  Elyada et al., 2019  Dominguez et al., 2019  Daviason et al., 2018 |
| CD146 | Pancreatic Cancer | Von Ahrens D et al., 2017 |
| IL6、CXCR7  MYLK、MYL9 | Esophageal carcinoma  Head and Neck Cancer | Qiao et al., 2017  Puram et al., 2017 |
