# Supplementary material for: Cancer associated fibroblast derived gene signature determines cancer subtypes and prognostic model construction in head and neck squamous cell carcinomas
Source: Cancer Med. 2022 Nov 20;12(5):6388–400. doi: 10.1002/cam4.5383 (PMC10028128; doi:10.1002/cam4.5383)
Supplement: Supplementary file 5 — Table S3 [file CAM4-12-6388-s002.docx]

**Supplementary Table 3 Characteristics of patients from GSE65858 data set**

| Characteristics | Cases（%） |
| --- | --- |
| Age  <65  ≥65 | 184（68.1%）  86（31.9%） |
| Gender  male  female  Tumor Site  oral  oropharynx  larynx  hypopharynx  unknown  Stage  I  II  III  IV  T Stage  T1  T2  T3 | 223（82.6%）  47（17.4%）  83（30.7%）  102（37.8%）  48（17.8%）  33（12.2%）  4（1.5%）  18（6.7%）  37（13.7%）  37（13.7%）  178（65.9%）  35（13.0%）  80（29.6%）  58（21.5%） |
| T4  N Stage  N0  N1  N2  N3  M Stage  M0  M1 | 97（35.9%）  94（34.8%）  32（11.9%）  132（48.9%）  12（4.4%）  263（97.4%）  7（2.6%） |
